# Selection for novel metabolic capabilities in *Salmonella enterica*

**DOI:** 10.1111/evo.13713

**Published:** 2019-03-22

**Authors:** Omar Warsi, Erik Lundin, Ulrika Lustig, Joakim Näsvall, Dan I. Andersson

**Affiliations:** ^1^ Department of Medical Biochemistry and Microbiology, Biomedical Center Uppsala University S‐751 23 Uppsala Sweden

**Keywords:** Carbon sources, experimental evolution, new metabolic functions, novel phenotypes

## Abstract

Bacteria are known to display extensive metabolic diversity and many studies have shown that they can use an extensive repertoire of small molecules as carbon‐ and energy sources. However, it is less clear to what extent a bacterium can expand its existing metabolic capabilities by acquiring mutations that, for example, rewire its metabolic pathways. To investigate this capability and potential for evolution of novel phenotypes, we sampled large populations of mutagenized *Salmonella enterica* to select very rare mutants that can grow on minimal media containing 124 low molecular weight compounds as sole carbon sources. We found mutants growing on 18 of these novel carbon sources, and identified the causal mutations that allowed growth for four of them. Mutations that relieve physiological constraints or increase expression of existing pathways were found to be important contributors to the novel phenotypes. For the remaining 14 novel phenotypes, whole genome sequencing of independent mutants and genetic analysis suggested that these novel metabolic phenotypes result from a combination of multiple mutations. This work, by virtue of identifying the genetic and mechanistic basis for new metabolic capabilities, sheds light on the properties of adaptive landscapes underlying the evolution of novel phenotypes.

Evolution of novel phenotypes is one of the central questions in evolutionary biology, playing a key role in major evolutionary transitions (Wong 1991; Szathmary and Smith 1995). These have been associated with ecological diversification (Blount et al. 2008; Wood and Erwin 2018) and with adaptive radiations (Warheit et al. 1999). An understanding of the mechanistic and genetic basis for the evolution of such novel functions, as well as of the associated trade‐offs, would give insights about the characteristics of the adaptive landscapes for these major transitions. To address this question, we used an experimental design to comprehensively explore the evolutionary potential for novel metabolic capabilities in a model organism, *Salmonella enterica* serovar Typhimurium strain LT2 (designated *S*. Typhimurium throughout the text), to utilize 124 different carbon compounds as sole carbon source (Fig. 1).

**Figure 1:**
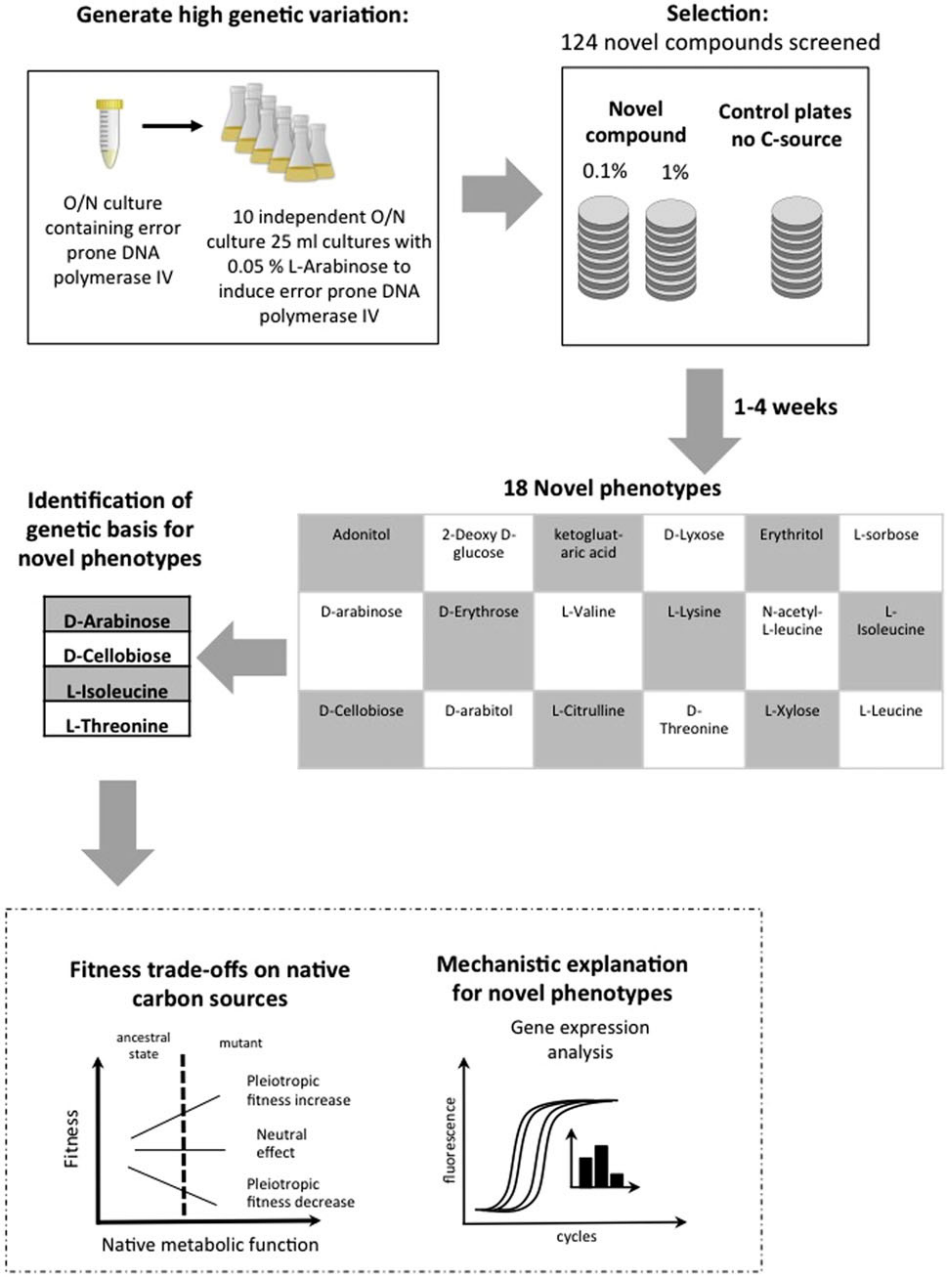
Selection of mutants with novel metabolic functions. An inducible error prone polymerase was used to increase the mutation rate of the ancestral *Salmonella* Typhimurium LT2 strain. The mutagenized population was spread on agar plates containing each of 124 novel carbon compounds. Mutants were selected, whole genome sequenced and causal mutations were identified. Fitness trade‐offs caused by the novel phenotypes were measured.

Microorganisms exhibit diverse metabolic functions (Barberán et al. 2014; Gilbert et al. 2014; Manoharan, et al. 2015; Anantharaman et al. 2016; Locey and Lennon 2016), where the interconnectedness of different biochemical pathways (Bhattacharya et al. 2003; Nielsen 2003; Fani and Fondi 2009) as well as secondary (promiscuous) activities of existing enzymes (Copley 2003; Nobeli et al. 2009; Khersonsky and Tawfik 2010) further increases an organism's metabolic repertoire. The underlying genetic architecture generating this complexity in biochemical pathways and functions has been well appreciated (Tavazoie et al. 1999; Jeong et al. 2000; Ravasz et al. 2002; Patil and Nielsen 2005; Deutscher et al. 2006). However, one outstanding question regarding these extensive biochemical pathways is how they can act as a source for the evolution of novel functions. The evolutionary potential of these metabolic pathways dictates the ability of these microorganisms to colonize new habitats and extend their ecological niches. This question has been important not only in the fields of ecology and evolution (Brown et al. 2004; Valentine 2007; Kearney et al. 2010; Wiedenbeck and Cohan 2011), but also in medical sciences, where it is important to understand how pathogens can acquire new mutations to colonize new niches within the human host (Sokurenko et al. 1999; Eisenreich et al. 2010; Bianconi et al. 2011; Rohmer et al. 2011; Fuchs et al. 2012).

The evolutionary potential to acquire novel metabolic functions depends not only upon the metabolic pathways present, but also on physiological constraints and functional trade‐offs that accompany these new phenotypes. By themselves, the study of constraints and trade‐offs accompanying adaptive evolution has been central in the field of evolutionary biology, both in eukaryotes (Mitchell‐Olds 1996; Etterson and Shaw 2001; Ghalambor et al. 2004; Shoval et al 2012) and in eubacteria (Mongold et al. 1996; Bohannan et al. 2002; Craig MacLean 2005; Duffy et al. 2006; Gudelj et al. 2007). A large number of studies have pointed out how these constraints and trade‐offs can limit the spectrum of adaptive mutations and evolutionary trajectories (Weinreich et al. 2005; Weinreich et al. 2006; Kvitek and Sherlock 2011; Salverda et al. 2011; Ito and Sasaki 2016). However, studies associating these constraints and trade‐offs with the evolution of novel phenotypes are limited (Downs 2006; Copley 2012; Chubiz and Marx 2017).

Another important, yet less observed scenario in the evolution of novel phenotypes, or generally during adaptive evolution, is when evolution proceeds by selection of those mutations that result in the disentangling of physiological constraints. Physiological constraints are here defined as the limitation to observe a given phenotype due to the underlying genetic organization or the interconnectedness of multiple phenotypes (Lande 1979; McDonald et al. 2009). Removal of these constraints becomes an important evolutionary route especially for novel metabolic functions since metabolic pathways are highly interconnected, with different metabolites effecting the regulation and functional state of any given protein. Despite this, one of the primary reasons for the limited evidence for such disentangling might be that this interconnectedness results in a limited number of possibilities to remove this constraint. As a result, our understanding of how mutations that get rid of these constraints can act as sources of novel phenotypes is also limited.

In this study, we generated mutagenized populations of *S*. Typhimurium and tested the ability of this mutagenized population to grow on 124 compounds that do not act as carbon source for this bacterium (Gutnick et al. 1969). Previous studies have generally explored the ability of native enzymes to perform novel metabolic activities across different organisms (Parker and Hall 1990; Hall 1999, 2003). Some early examples of these studies include the ability of *Escherichia coli* to metabolize cellobiose through mutations in the *cel* operon (Parker and Hall 1990), the ability of a *E. coli* with a deletion in the *lac* operon to metabolize lactose through mutations in the *ebg* operon (Hall 1999, 2003), the ability of *Klebsiella pneumoniae* to metabolize xylitol through mutations in the ribitol pathway (Mortlock and Wood 1971; Rigby et al. 1974), and the ability of *Pseudomonas aeruginosa* to metabolize butyramide through mutations in the aliphatic amidase pathway (Brown et al. 1969).

Our results show that *S*. Typhimurium acquired the ability to grow on a normally nonutilizable carbon source for at least 14% (18/124) of the compounds tested. We identified the causal mutations for four of these novel phenotypes and showed that mutations resulting in regulatory changes and which relieved physiological constraints contributed to the evolution of novel phenotypes.

## Materials and Methods

### MUTAGENESIS APPROACH AND CARBON COMPOUNDS USED

*S*. Typhimurium containing the pSMP24 (pBAD::*dinB* Maisnier‐Patin et al. 2005) plasmid was used as the starting strain for these experiments. The pSMP24 plasmid carries the error prone DNA IV polymerase under the control of a L‐arabinose inducible promoter. From an overnight culture, 10^3^ cells were used to start each of ten 25 mL cultures. The media used for these ten cultures was Lysogeny broth (LB) with 0.05% L‐arabinose. After an overnight growth, each of these 25 mL cultures were washed thrice with phosphate‐buffered saline (PBS) and were re‐suspended in 5 mL of PBS. Hundred microliter of this bacterial suspension (corresponding to about 2.5 × 10^9^ cells) was then spread on minimal media plates containing different carbon compounds as the sole carbon source (10 plates for each carbon source). The list of carbon compounds used was based on earlier work done by Gutnik et al. (1969). A total of 124 carbon compounds (Table S1) was used at two different concentrations (0.1% and 1% [w/v]). Fluctuation tests were performed using selection for rifampicin resistance as a proxy to measure the increase in mutation rate caused by the error prone polymerase (Table S2). Plates were incubated for 2–4 weeks at 37°C, and were checked for growth every 7 days.

### P22 TRANSDUCTION AND ISOLATION OF MUTATIONS CONFERRING NOVEL METABOLIC FUNCTIONS

To identify single mutations that caused the novel phenotypes a P22 transduction protocol was used. P22 lysate was generated on each of the clones that were able to use a novel carbon compound as a sole carbon source in minimal media. Lysates were generated by mixing 1 mL of P22 stock (∼1 × 10^6^ pfu/mL) and 200 μL of overnight grown donor strain in a 10 mL tube, which was then incubated overnight with shaking at 37°C. The next day 100 μL of chloroform was used to kill surviving cells, after which the mixture was spun down, and the supernatant was used as the lysate. To obtain transductants, 1 μL of this lysate was mixed with 100 μL of overnight grown wild‐type *S*. Typhimurium strain and incubated with shaking at 37°C for 30 minutes. The mixture was then washed with PBS twice and then plated on minimal media plates with the appropriate carbon sources. Plates were generally incubated for 2–4 weeks. Control plates with nontransduced wild‐type *S*. Typhimurium cells and P22 lysate alone were also plated individually. P22‐free transductants were identified by streaking on Evans Blue Uranine (EBU) plates.

### WHOLE GENOME SEQUENCING AND SEQUENCE ANALYSIS

Mutations that could contribute to the novel phenotypes were identified by whole genome sequencing. DNA was extracted from 1 mL overnight cultures using the MasterPure Complete DNA & RNA Putification Kit (Epicentre) according to the manufacturer's instructions. Illumina's Nextera XT kit was used to make libraries (2 × 300) to be sequenced with Miseq. Samples were dual‐indexed and pooled together. Average whole genome coverage per sample was 30X. Analysis of the fastq files obtained from Miseq sequencing was performed using CLC genomics Workbench version 8 and were mapped to the reference genome of the ancestral *S*. Typhimurium strain. SNP calling and structural rearrangements were both assessed using this tool.

### MEASUREMENT OF EXPONENTIAL GROWTH RATES AND FITNESS TRADE‐OFFS

To determine fitness trade‐offs for mutants able to grow on L‐isoleucine and L‐threonine as the sole carbon source, exponential growth rates were measured using a BioscreenC analyzer at OD_600_, with measurements taken every 4 minutes. KaleidaGraph software was used to calculate the maximum exponential growth rate from the OD_600_ data, using the optical density (OD) values between 0.02 and 0.09. Overnight cultures were diluted 1:1000 fold in appropriate media. Growth rates for mutants able to grow on L‐isoleucine as sole carbon source was measured on minimal media containing 0.2% of different carbon sources (acetate, glycerol, citrate, or glucose). Growth rates for mutants able to grow on L‐threonine as sole carbon source was measured on 0.1% glucose minimal media, with and without 0.1% L‐threonine or 0.1% L‐leucine. For each clone, measurements were performed on four biological replicates. In each run, the exponential growth rate for the ancestral strain was also measured. Relative exponential growth rate was calculated as the ratio between the exponential growth rate of the clone and that of the ancestral strain. Error bars represent standard deviation. Two‐tailed Student's *t*‐test was performed to calculate significance.

### qPCR MEASUREMENTS OF EXPRESSION OF GENES INVOLVED IN THE ISOLEUCINE BIOSYNTHESIS PATHWAY AND GENES FROM THE THREONINE DEGRADATION PATHWAY

For qPCR, cells were grown to OD_600_ = 0.3 (measured with 1 cm light path in a Shimadzu UV mini 1240 spectrophotometer) in 10 mL of either 0.1% glucose M9‐minimal media, 0.1% glucose M9‐minimal media supplemented with 0.1% of L‐isoleucine, or 0.1% glucose M9‐minimal media supplemented with 0.1% of L‐threonine. RNA was extracted from 500 μL of the cultures, using the RNeasy Mini Kit (Qiagen) as per the manufacturer's protocol, and DNase treated using the Turbo DNA‐free kit (Ambion) according to the manufacturer's protocol. The DNA‐free RNA was run on a 1% (w/v) agarose gel for visual inspection. Five hundred nanograms of RNA (quantified using the Qubit RNA BR assay kit) was used for cDNA preparation using the High Capacity Reverse Transcription Kit (Applied Biosystems). RT‐qPCRs were performed using the PerfecTa Sybr Green SuperMix (Quanta Biosciences). The house keeping genes used as references were *cysG* and *hcaT*. Three biological replicates and three technical replicates were used in each case. The averages are based on biological replicates. Error bars represent standard deviation. Two‐tailed Students‐*t* test was performed to test for statistically significant differences.

## Results

### SELECTION OF *S*. TYPHIMURIUM MUTANTS THAT CAN GROW ON NORMALLY NONUTILIZABLE CARBON SOURCES

The potential carbon sources used for selection in our study were a subset of the list generated by Gutnik et al. (1969). Out of the 600 compounds tested in that study, we selected those carbon sources that did not contain phosphate groups and were not polymers with the reasoning that they might be poorly taken up by bacterial cells. In total, we chose 124 compounds that were previously shown to not act as carbon sources for *S*. Typhimurium (Table S1), and two concentrations of the carbon source (0.1% and 1%) were used for the selection experiment. A wild‐type *S*. Typhimurium strain was transformed with a plasmid that carried the L‐arabinose inducible error prone polymerase DinB (pSMP24), and was used as the ancestral strain in our experiments. Presence of an inducible DinB allowed us to increase the mutation rate by 400‐fold (Maisnier‐Patin et al. 2005) and hence sample greater genetic variation. We then performed the selection with ∼10^10^ bacteria per concentration of each compound. Mutants able to grow on 18 carbon sources were obtained (Table S1, Fig. 1). Broadly these 18 carbon sources can be divided into six different classes: amino acids (7), monosaccharides (6), sugar alcohols (3), disaccharides (1), and a ketoacid (1).

### WHOLE GENOME ANALYSIS AND IDENTIFICATION OF CAUSAL MUTATIONS THAT ALLOW GROWTH ON NOVEL CARBON SOURCES

Given the DinB‐mutagenesis approach that was used to generate mutants with novel phenotypes, we expected that each mutant contained several mutations. Thus, to identify the causal mutation allowing growth on the novel carbon source, a P22 transduction protocol was used. For the P22 transduction experiments, lysates were generated on each mutant, which was then used for transduction of the ancestral *S*. Typhimurium strain. Transductants were selected on appropriate carbon sources and mutants that grew were candidates for having received the causal mutation. Whole genome sequencing of the transductants was performed that allowed us to identify the responsible mutations in four out of the 18 mutants (Table 1). Mutations identified by this method involved mutations in gene *celD* (allowing for growth on D‐cellobiose as sole carbon source), mutations in gene *ilvN* (allowing for growth on L‐isoleucine as sole carbon source), mutations in genes *lrp* and *kbl* (allowing for growth on L‐threonine as sole carbon source) and *fucR* (allowing for growth on D‐arabinose as sole carbon source) (Table 1).

**Table 1:** Causal mutations for growth on new carbon sources

Strain	Novel carbon source supporting growth	Gene causing novel phenotype	Amino acid change/mutation
A56875	D‐cellobiose	*celD*	N238S
DA58010	L‐isoleucine	*ilvN*	N35K
DA58011*	D‐arabinose	*fucR*	S75R
DA62190	L‐threonine	*lrp*	A63E
DA62191	L‐threonine	*kbl*	–60 G>A

Causal mutations allowing growth on novel carbon sources were identified by a P22 transduction protocol and by whole genome sequence analysis.

*only locally sequenced at the *fucR* gene.

Whole genome sequencing was also performed on mutants that were able to grow on four different carbon sources, but where P22 transductions did not produce any recombinants capable of growth. These included the carbon sources L‐valine (one mutant), 2‐Deoxy‐D‐glucose (three mutants), D‐adonitol (one mutant), and D‐erythrose (two mutants) (Table S3). In a majority of these mutants, we observed at least nine nonsynonymous mutations suggesting that multiple mutations are required to generate the new phenotype (which was also supported by the fact that no transductants that could grow on the novel carbon sources were obtained). In cases where more than one independent mutant was sequenced for a given carbon source (2‐Deoxy‐D‐glucose and D‐erythrose), there was also no overlap with regards to, which genes had mutations, nor any overlap in the biochemical pathways that these genes belonged to; indicating that for these carbon sources multiple mutational pathways exists that allow generation of the novel phenotype (Table S3). For mutants growing on L‐valine and D‐adonitol as the sole carbon source, whole genome sequencing did not reveal any obvious targets.

Mutants able to metabolize D‐cellobiose as the sole carbon source had mutations in the transcriptional regulator *celD*, which is homologous to the *celR* transcriptional regulator of the cryptic *cel* operon in *E. coli*. Besides the mutation that we obtained by the P22 transduction strategy (*celD* Asn238Ser), whole genome sequencing of other mutants that could utilize D‐cellobiose revealed several different mutations in gene *celD* that could potentially result in growth of the mutant on D‐cellobiose as sole carbon source (Table S4). Mutants able to metabolize D‐arabinose had a mutation in the transcriptional regulator *fucR*. Mutations in this gene results in D‐arabinose to act as an inducer for the L‐fucose degradation pathway. Expression of enzymes from this pathway allows for utilization of D‐arabinose as the sole carbon source (Zhu and Lin 1986). Since mutations in both these genes and the underlying mechanisms generating the novel phenotypes have been described earlier (Zhu and Lin 1986; Parker and Hall 1990), they will not be discussed further here.

### MUTATIONS in *ilvN* ALLOWS FOR USAGE OF L‐ISOLEUCINE AS SOLE CARBON SOURCE BY REMOVAL OF A PHYSIOLOGICAL CONSTRAINT

**Figure 2:**
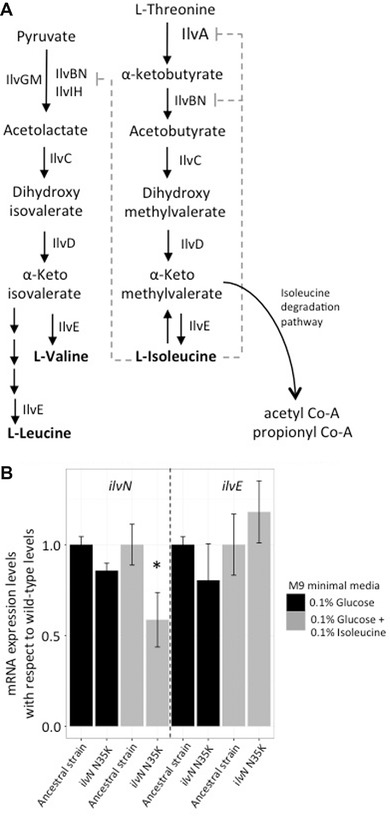
Utilization of L‐isoleucine as sole carbon source. (A) Common enzymes involved in biosynthesis of L‐isoleucine, L‐leucine and L‐valine: Pathway showing common enzymes for synthesis of branched chain amino acids L‐isoleucine, L‐valine, and L‐leucine. All amino acids exert feedback inhibition on these common enzymes. Isoleucine can also enter the isoleucine degradation pathway. (B) Gene expression analysis for genes in *ilvN and ilvE*: qPCR measurements of genes *ilvN* and *ilvE* in wild‐type ancestral strain and the IlvN N35K mutant in different media (shown as different colored bars). The values are normalized to the expression levels observed in the wild‐type strain when grown in the respective media. Error bars represent standard deviation. Two‐tailed Student's *t* test was used to calculate significant differences (denoted by * when applicable). No difference was observed in expression level of *ilvE* gene in different media conditions.

Mutants that grew on L‐isoleucine as sole carbon source had mutations in the *ilvN* gene. Two unique mutations were observed: N35K and V36A. *ilvN* and *ilvB* encode the two subunits of acetolactate synthase 1, one of the three isozymes that catalyze the second step of L‐isoleucine biosynthesis and the first step of L‐valine and L‐leucine biosynthesis (Fig. 2A). Both the mutations found in the IlvN protein are in positions that have been predicted to interact with L‐valine for feed‐back inhibition of the biosynthetic pathway (Kaplun et al. 2006). It is thus likely that these mutations affect the interaction of IlvN with L‐isoleucine as well. We hypothesized that the amino acid substitutions in IlvN either lead to loss of feed‐back inhibition (at the level of translation) by L‐isoleucine, preventing the starvation of the cells for L‐valine and L‐leucine, or results in an increased expression of genes in the L‐isoleucine degradation pathway (Fig. 2A). The isoleucine degradation pathway allows for the conversion of L‐isoleucine to acetyl‐CoA that can be channeled into other metabolic pathways.

**Figure 3:**
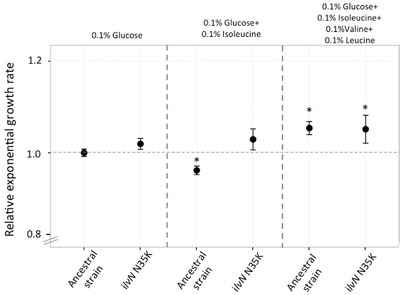
Isoleucine toxicity and rescue of ancestral strain in presence of valine/leucine. Relative exponential growth rates of the llvN N35K mutant compared to the wild‐type strain in different media: 0.1% minimal glucose media, 0.1% minimal glucose media with 0.1% isoleucine and 0.1% minimal glucose media with 0.1% each of isoleucine, valine, and leucine. Error bars represent standard deviation. Two‐tailed Student's *t* test was used to calculate significant differences at *P* < 0.01 (denoted by * when applicable).

To differentiate between these two hypotheses, we measured the expression levels of the *ilvN* and *ilvE* genes in the wild‐type strain and the *ilvN* N35K mutant, in 0.1% glucose minimal media and in 0.1% glucose minimal media with 0.1% isoleucine (Fig. 2B). The *ilvE* gene is responsible for the last step of isoleucine/valine/leucine biosynthesis as well as the first step of the isoleucine degradation pathway. No difference between the expression levels of *ilvE* in the wild‐type strain or the *ilvN* N35K mutant was observed (Fig. 2B), supporting the hypothesis that utilizing L‐isoleucine as carbon source only requires the cell to overcome valine and/or leucine starvation. To further demonstrate that excess L‐isoleucine causes L‐valine starvation, and that this starvation is removed in the *ilvN* N35K mutant, we measured growth rates of wild‐type and the *ilvN* N35K mutant under three different growth conditions: minimal media with 0.1% glucose, minimal media with 0.1% glucose and 0.1% isoleucine and minimal media with 0.1% glucose and 0.1% each of L‐valine, L‐leucine, and L‐isoleucine. As expected the growth rate of the wild‐type strain was lower in the environment where only L‐isoleucine was present, but no reduction was seen for the *ilvN* N35K mutant in this media (Fig. 3). Also, this reduction was no longer seen for the wild‐type strain or the mutant strain in the presence of L‐valine, L‐isoleucine, and L‐leucine (Fig. 3). These results demonstrate an example of evolution of a new phenotype by means of overcoming a physiological and regulatory constraint.

### MUTATIONS IN THE GENE *lrp* AND UPSTREAM OF GENE *kbI* INDIVIDUALLY ALLOW UTILIZATION OF L‐threonine AS SOLE CARBON SOURCE BY INCREASING THE FLUX THROUGH THE L‐threonine DEGRADATION PATHWAY

**Figure 4:**
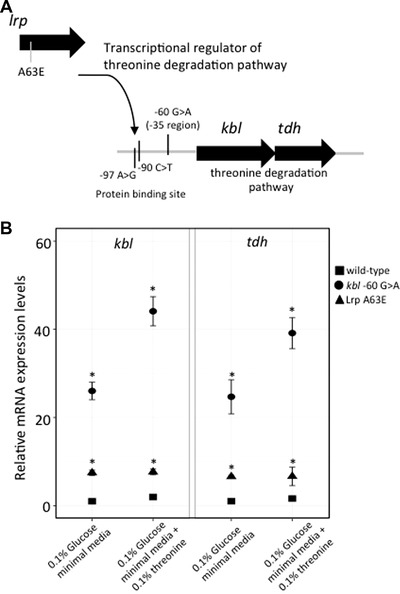
Mutations allowing utilization of L‐threonine as sole carbon source. (A) Mutations in *lrp* and upstream of *kbl* allowing utilization of L‐threonine as sole carbon source. (B) Gene expression analysis for genes *kbl* and *tdh* from threonine degradation pathway: qPCR measurements of genes *kbl* and *tdh* in Lrp A63E and *kbl* ‐60 G>A mutants in different media. The values are normalized to the expression levels observed in the wild‐type strain when grown in 0.1% glucose M9‐minimal media. Error bars represent standard deviation. Two‐tailed Student's *t* test was used to calculate significant differences at *P* < 0.01 (denoted by * when applicable).

Mutations affecting two different genes allowed for growth on L‐threonine as sole carbon source. The first mutation was seen in gene *lrp* resulting in a nonsynonymous change (A63E; Fig. 4A). The Lrp protein is a transcriptional regulator that induces the expression of genes belonging to the L‐threonine degradation pathway. Mutations in *lrp* could thus potentially result in increased expression of enzymes from this pathway, allowing for sufficient amounts of acetyl‐CoA to be produced for cell‐growth. Increased expression of enzymes from this pathway has been shown to allow usage of L‐threonine as a sole carbon source in *E. coli* (Chan and Newman 1981). The second mutation that allows growth on L‐threonine as sole carbon source was observed upstream of gene *kbl* (–60 G>A) in the –35 element of the promoter. The product of this gene catalyzes the second step of threonine dehydrogenase mediated threonine degradation pathway. Consequently, the observed upstream mutation in this gene could also act by increasing the expression of genes involved in threonine degradation pathway (Fig. 4A). Whole genome sequencing was also performed on other mutants that could grow on L‐threonine as a sole carbon source. Mutants with different mutations upstream of gene *kbl* were identified (Fig. 4A). Besides the mutation observed in the –35 element of the promoter, two other mutations upstream of the gene *kbl* were observed in LRP‐binding sites.

Thus, it is likely that these mutations allow usage of L‐threonine as a sole carbon source by increasing the flux through the threonine degradation pathway. We supported this hypothesis by measuring mRNA expression levels for genes *kbl* and *tdh* (that are part of the threonine degradation pathway) in both the *lrp* A63E and *kbl* –60 G>A mutants. Expression levels were measured in 0.1% glucose M9‐minimal media, and in 0.1% glucose M9‐minimal media supplemented with 0.1% L‐threonine. In both environments upregulation of these genes was observed, as compared to the expression levels in the wild‐type strain (Fig. 4B). The upregulation observed for the *lrp* A63E mutant was similar across both the environments (∼7X). The upregulation in the *kbl* –60 G>A mutant was higher as compared to the *lrp* A63E mutant in 0.1% glucose minimal media (∼24X), which further increased in the presence of 0.1% L‐threonine (∼40X).

### FITNESS TRADE‐OFFS BETWEEN NOVEL AND ANCESTRAL FUNCTION OF *ilvN, lrp, AND kbl*

**Figure 5:**
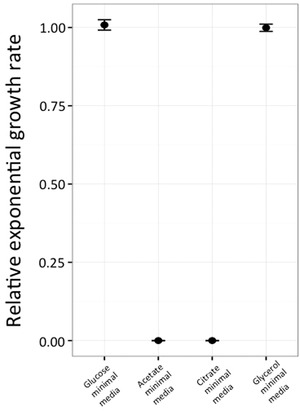
Relative exponential growth rates for llvN N35K mutant on alternate native carbon sources. Exponential growth rates of the mutants were measured and normalized with respect to growth rate of wild‐type strain in minimal media containing 0.2% of different carbon sources (glucose, acetate, citrate, or glycerol).

To elucidate whether the ability to utilize a new carbon source was associated with a loss of the ancestral function, we measured exponential rates for mutants that grew either on L‐isoleucine or L‐threonine as a sole carbon source. It has been demonstrated that the *ilvBN* system is essential for growth on poor carbon sources (deletion of this system results in inability to grow), and under these conditions it becomes the important route for isoleucine biosynthesis (Dailey and Cronan 1986). To test if this function was compromised in the IlvN N35K mutant, we measured growth rates of the mutant on minimal media with glucose, acetate, glycerol, or citrate as sole carbon sources (Fig. 5). Our results demonstrate that the mutation N35K has drastic effects on the native function of IlvN, as shown by its inability to grow on both acetate and citrate as sole carbon source. However, no effect on growth rate was observed in minimal media with glycerol as sole carbon source.

**Figure 6:**
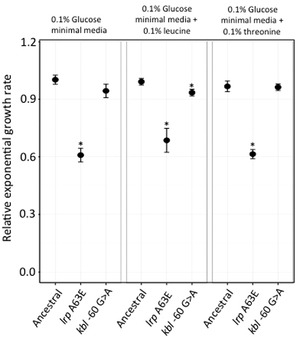
Fitness trade‐offs observed in Lrp A63E and *kbl* ‐60 G>A mutants under different growth conditions. Relative exponential growth rate for Lrp A63E and *kbl* ‐60 G>A mutants, with respect to the wild‐type strain, measured in different media: 0.1% minimal glucose media, 0.1% minimal glucose media with 0.1% threonine and 0.1% minimal glucose media with 0.1% leucine. Two‐tailed Student's *t* test was used to calculate significant differences at *P* < 0.01 (denoted by * when applicable).

The Lrp protein is a leucine‐responsive regulatory protein that senses leucine concentration in the environment and regulates genes involved in feast‐famine functions (Tani et al. 2002). To investigate if the amino acid change A63E in Lrp that confers a novel phenotype of utilizing L‐threonine as sole carbon source results in functional trade‐offs, growth rates for this mutant was measured in glucose minimal media, and in glucose minimal media supplemented with either 0.1% leucine or 0.1% threonine. Under all three conditions, a reduction of growth rate (30–40% across the different media) was observed for the mutant as compared to the ancestral strain (Fig. 6).

Multiple mutations were also observed in the promoter region of gene *kbl*, which catalyzes the second step in the threonine degradation pathway. To investigate if these mutations caused trade‐offs in alternate environments, a P22 transductant with only the mutation at –60 G>A position in the promoter region was chosen and growth rates were measured in three different environments: glucose minimal media, and in 0.1% glucose minimal media supplemented with 0.1% L‐leucine or 0.1% L‐threonine. A reduction of growth rate for the *kbl* –60 G>A mutant, as compared to the wild‐type strain, was observed in glucose minimal media supplemented with 0.1% L‐leucine (*P* = 0.0037). In glucose minimal media only a small reduction in growth rate for the mutant was observed with respect to the wild‐type strain (*P* = 0.032), while no difference was observed in glucose minimal media supplemented with 0.1% L‐threonine (Fig. 6).

## Discussion

### DIFFERENT MUTATIONAL TRAJECTORIES RESULT IN GAIN OF NOVEL METABOLIC FUNCTIONS

Our experimental design allowed us to ask if and how an organism can mutationally gain new metabolic functions to utilize new resources, and thus contributes to our understanding of adaptive landscapes for novel phenotypes. The mutagenesis approach combined with the large population sampling size allowed us to test every single SNP in the genome and a fraction of combinations of SNPs for their ability to allow growth on novel carbon sources. We found that *S*. Typhimurium could metabolize as much as 14% (18/124) of the compounds tested and for four different compounds (D‐cellobiose, L‐isoleucine, L‐threonine, and D‐arabinose) we could identify single mutations that conferred the novel phenotype. In the remaining cases, for two different carbon sources (2‐Deoxy‐D‐glucose and D‐erythrose), where multiple independent clones were whole genome sequenced, there was no overlap in the genes in which mutations were found. This observation suggests that for these novel phenotypes, several genetically different routes are present that allow the ability to metabolize a given carbon source. Furthermore, whole genome sequencing of mutants that could grow on four other carbon sources (L‐valine, 2‐Deoxy‐D‐glucose, D‐adonitol, and D‐erythrose) and where P22 transduction did not work suggest that multiple mutations contribute to their novel phenotypes. Obviously we cannot rule out the possibility that some mutations (amplifications or insertions) were not picked up in our experiments because of their instability and possible reversion to the ancestral state, which could be the case with gene amplifications.

### OVERCOMING PHYSIOLOGICAL CONSTRAINTS FOR ACQUISITION OF NEW METABOLIC PHENOTYPES

Adaptation to new environments can be limited due to physiological constraints (Gould and Lewontin 1979; Galis and Metz 2007), suggesting that mutations that overcome such constrains could allow evolution of new functions (Wagner and Muller 2002). One such case observed in our study was the ability to use L‐isoleucine as the sole carbon source. L‐isoleucine can be degraded in *S*. Typhimurium to acetyl‐CoA that can enter other pathways for generation of carbon compounds. However, L‐isoleucine is known to not serve as a carbon source, most likely because L‐isoleucine shares its biosynthesis enzymes with other branched chain amino acids, L‐valine and L‐leucine, and excess of L‐isoleucine in the environment would lead to feedback inhibition of these enzymes, resulting in starvation for L‐valine and L‐leucine. Similar effects are seen when excess L‐valine is present in the growth medium, where starvation for L‐isoleucine is observed (Temple et al. 1965). Mutations in the gene *ilvN* allow the mutants to grow on L‐isoleucine as the sole carbon source. These mutations were observed in two adjacent positions on this protein (N35K and V36A), both of which have been predicted to interact with L‐valine, potentially causing feedback inhibition (Kaplun et al. 2006). Thus, the observed mutations could result in reduced interaction between IlvN protein and the L‐isoleucine in the medium, thus overcoming L‐valine or L‐leucine starvation. Alternatively, this mutation could also result in lowering the feedback repression from the L‐valine being synthesized in the cell, having the same outcome of avoiding L‐valine starvation. These results were corroborated by our qPCR results suggesting that it was sufficient to overcome this constraint to allow usage of L‐isoleucine as carbon source. The interconnectedness is an inherent part of many metabolic pathways and results in physiological constraints. Our results suggest that mutations that relieve these physiological constraints might play an important role in evolution of new phenotypes.

### FITNESS TRADE‐OFFS ACCOMPANYING NOVEL PHENOTYPES

Besides physiological constraints, trade‐offs between new and old capabilities can also influence the evolution of new phenotypes. *ilvN* mutants were unable to grow in either acetate or citrate as sole carbon source. Despite the presence of other IlvN‐like isozymes in the cell, IlvN plays an important role in synthesis of branched chain amino acids when the bacteria are growing on poor carbon sources (defined as carbon sources that confer slow growth), like acetate and citric acid. The inability of the *ilvN* mutant to grow on these poor carbon sources represents a functional trade‐off of the IlvN protein that results in loss of the native function of the protein (Dailey and Cronan 1986). Unexpectedly though the cells were able to grow on minimal media containing glycerol as the sole carbon source, which is another poor carbon source. This suggests that these functional trade‐offs observed are specific in nature, highlighting another level of complexity in understanding the evolution of the new phenotypes. Reduction in growth rate was also observed for the Lrp A63E mutant that allowed growth on L‐threonine as sole carbon source. This trade‐off is expected since it has been shown that Lrp regulates close to 400 genes in *E. coli* (Tani et al. 2002). Thus, mutations in this gene would have significant pleiotropic effects, which can dictate the evolution of the novel phenotype.

In conclusion, our study provides an analysis of how novel metabolic functions can arise from one or a few point mutations. Our results show how mutations that affect regulation play a significant part in adaptive evolution of novel phenotypes, and how mutations that remove physiological constraints can aid in evolution of novel phenotypes. However, our study also highlights the limitation of evolution of novel phenotypes by single‐step mutations under hard selection on novel carbon sources, given that we observed mutants for only 14% of the carbon sources tested. Future studies will focus on generation of novel phenotypes using an experimental evolution approach of evolving populations in the presence of utilizable yet poor carbon sources supplemented with a potential nonutilizable carbon source. This strategy might allow an expanding population to accumulate multiple mutations to allow efficient utilization of the novel carbon source.

## CONFLICT OF INTEREST

The authors report no conflict of interest.

Associate Editor: T. Cooper

Handling Editor: Mohamed. A. F. Noor

## Supporting information


**Table 1**. Carbon compounds used for selection of mutants with novel metabolic capabilities

**Table 2**. Increased mutation rates obtained using an error‐prone polymerase

**Table 3**. Mutations observed in mutants growing on novel carbon sources (where causal mutation is not identified)

**Table 4**. Mutations in gene *celD* that potentially allow for growth on cellobiose as sole carbon source
