# Supplementary material for: Selection for novel metabolic capabilities in Salmonella enterica
Source: Evolution. 2019 Mar 22;73(5):990–1000. doi: 10.1111/evo.13713 (PMC6593847; doi:10.1111/evo.13713)
Supplement: Supplementary file 4 — Table 4. Mutations in gene celD that potentially allow for growth on cellobiose as sole carbon source [file EVO-73-990-s004.docx]

| **Strain** | **Non-synonmous mutations in gene *celD*** |
| --- | --- |
| DA37545 | Met236Arg |
| DA37546 | Tyr30Cys |
| DA37547 | Val132Gly |
| DA37548 | His182Asp |
| DA48862 | Asn238Ser |
| DA56875*  (Obtained by P22 transduction strategy) | Asn238Ser |

Supplementary table 4: Mutations in gene *celD* that potentially allow for growth on cellobiose as sole carbon source
